# Supplementary material for: Development of a miRNA-Based Model for Lung Cancer Detection
Source: Cancers (Basel). 2025 Mar 10;17(6):942. doi: 10.3390/cancers17060942 (PMC11940216; doi:10.3390/cancers17060942)
Supplement: Supplementary file 1 [file cancers-17-00942-s001.zip › cancers-3490179-supplementary.pdf]

Supplementary Table and Figures

miRNA	miRNA expression from prior research	miRNA expression in discovery cohort	Potential role in lung cancer	Citation
miR-1246	Up	Up	Stemness and invasiveness, downregulation of tumour suppressor gene BTG3 and cell apoptosis	[42, 45, 67, 68]
miR-1290	Up	Up	Stemness and Invasiveness	[42, 45, 69]
miR-221-3p	Up	Up	Targets p27 and promotes cellular growth and invasiveness	[34, 64, 70, 71]
miR-223-5p	Up/Down	Up	Control cellular growth and invasiveness	[64, 72-74]
miR-1268b	Up	Up	Increased expression in resected (early) lung cancer	[75, 76]
miR-106b-5p	Up	Up	Promotes proliferation and inhibits apoptosis by regulating BTG3 and downregulating IGSF 10	[43, 77]
miR-26b-5p	Up	Up	Promotes proliferation and through downregulation of DUXAP8 and metastasis through MIEN1	[34, 78-80]
MIR-21-5p	Up	Up	Promote non-small cell lung cancer migration and invasion by downregulating SOCS1, SOCS6, and PTEN	[81, 82]
miR-196a-5p	Up	Up	Promotes proliferation and invasion through targeting	[38-40]

			HOXA5 and PI3K/AKT pathway. Stemness through down-regulation of GPX3	
miR-130b-5p	Up	Up	Stemness through inverse regulation of DPP-4 pathway and invasiveness up upregulating metalloproteinase-2	[41, 83]
miR-155-5p	Up	Up	Promote non-small cell lung cancer progression by downregulating SOCS1, SOCS6, and PTEN. Target HBP1, TJP1, SMAD5 and PRKAR1A which are involved in the oxidative stress process of lung cancer.	[81, 84]
miR-23a-3p	Up	Up	Increases angiogenesis and vascular permeability	[85, 86]
miR-16-5p	Up/Down	Up	Promotes proliferation and invasion through PI3K/AKT pathway.	[70, 87]
miR-222-3p	Up	Up	Promotes Cell Proliferation and Inhibits Apoptosis by Targeting PUMA (BBC3)	[88, 89]
miR-451a	Up/down	Up	Tumor suppressor, positive relationship between miR-451a and DOX sensitivity in lung cancer cells	[25, 90]
miR-486-5p	Down	Up	Tumor suppressor , Suppresses Cell Growth With the Involvement of a Target PIK3R1	[34, 70, 91]

miR-25-5p	Up	Not Expressed	Stemness through LATS2/YAP signalling pathway	[42, 92]
miR-630	Up/ Down	Not Expressed	Stemness, suppresses non-small cell lung cancer by targeting vimentin	[42, 93]
miR-130a-5p	Down	Not Expressed	Stemness, microRNA-130a-5p/RUNX2/STK32A network modulates tumor invasive and metastatic potential	[42, 94]
miR-30b-3p	Up/ Down	Not Expressed	Inhibits cell invasion and migration through targeting collagen triple helix repeat	[34, 70, 95]
miR-141-5p	Up	Not Expressed	Promotes cell invasion and proliferation by targeting KLF9	[96, 97]
miR-449a	Down	Not Expressed	Tumour Suppressor	[28, 98]
miR-638	Down	Not Expressed	DNA damage repair, inhibit invasion and proliferation of lung cancer	[99, 100]
miR-6075	Up	Not Expressed	Tumor Oncogene	[75, 101]
miR-9-5p	Up	Not Expressed	Proliferation, metastasis and invasion by NSCLC cell through downregulation of TGFBR2	[102, 103]

Supplementary Table S1. miRNA biomarker selection

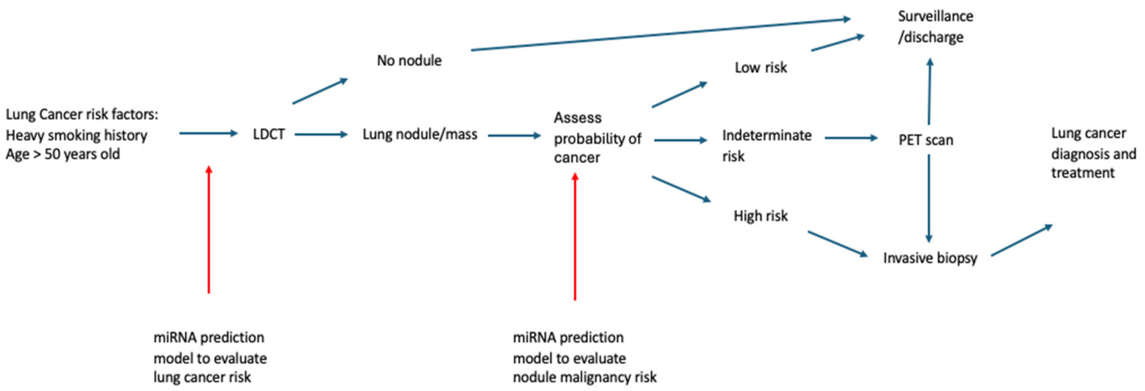

Supplemtary Figure S1. Use of miRNA-based prediction model to improve lung cancer screening

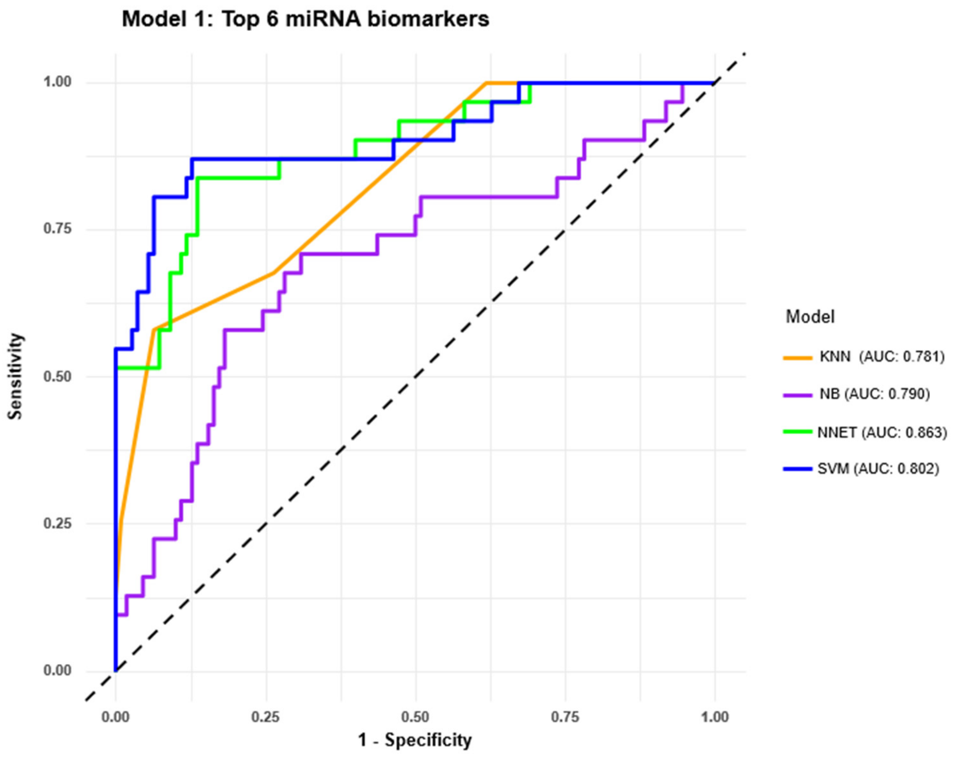

Supplementary Figure S2. ROC curves for Model

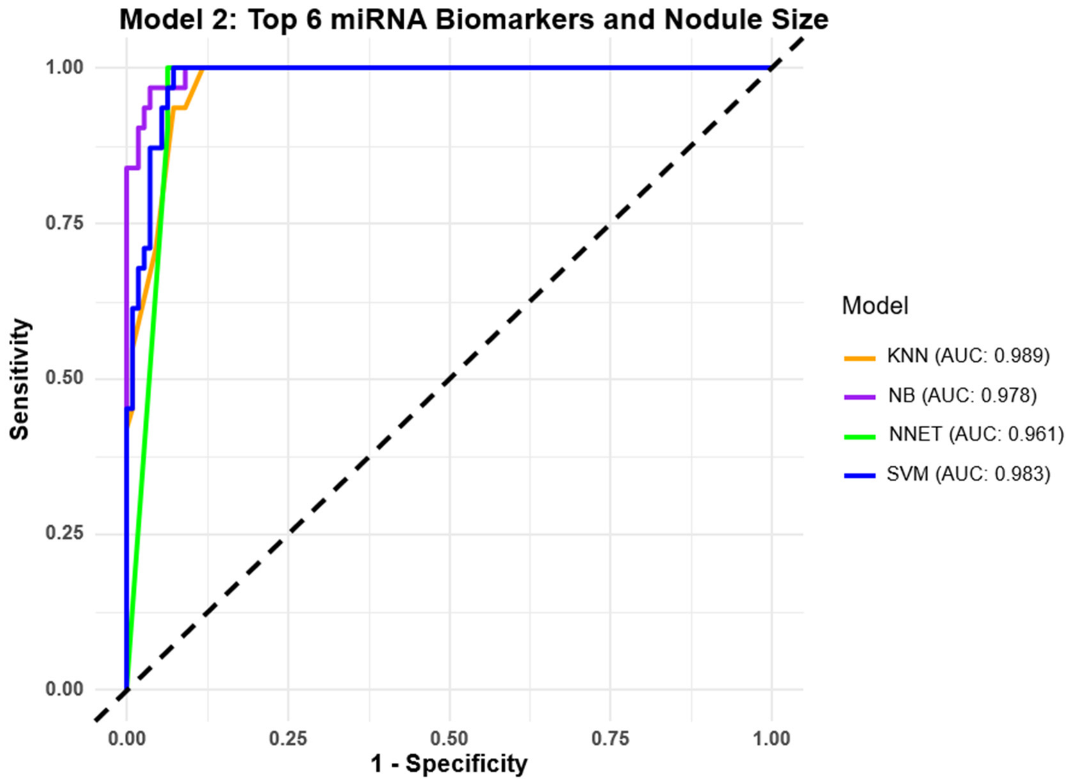

Supplementary Figure S3. ROC curves for Model 2.
